# Progress in Diseases of the Blood

**Published:** 1901-11-23

**Authors:** 


### Progress in Diseases of the Blood.

**Leucæmia.** — The combination of myelogenous leucæmia with phthisis is exceedingly rare. In a case recorded by Elsner and Groat,[1] the patient was a male, aged 40. He first complained of weakness and an enlargement of the abdomen, but after several months a cough and night sweats appeared. Both lungs and the larynx were affected, the spleen was of enormous size, and the blood contained a great number of myelocytes, about three million red cells with a few megalo-blasts, 320,000 leucocytes, many mast cells and eosinophiles. As the lung trouble advanced the leucocytes decreased in number, the reduction of the myelocytes being marked, while the lymphocytes and polynuclear cells increased. In most of the recorded cases where some other infective disease accompanied the leucæmia there has been a reduction of the leucocytes after the second infection. With regard to the lungs lymphoid infiltration may take place and be mistaken for tuberculous disease, but here the changes were definitely tuberculous and the usual bacilli were found. Another instance is reported by Sturmdorf[2] in a married woman of 35, who presented very similar physical signs to those seen in the last case. While she was under observation, when febrile temperatures occurred, there was a fall in the myelocytes and a rise in the polynuclear cells, but the numbers and proportion never became anything like normal. The lung changes were shown to be tuberculous, and not merely the result of lymphoid deposits. Saundby remarks that occasionally recovery from splenic leucæmia takes place, and gives an instance where a patient was found to be in perfect health six years after the disease was diagnosed, and occasionally, from some cause or other, the leucocytes may become normal though the disease is by no means cured. Apparently some such effect is produced by a febrile infection, though without any necessary tendency towards complete recovery.

**Lymphatic Leucæmia.**—Fränkel laid down that acute leucæmia is always lymphatic in type and is accompanied by the presence of large lymphocytes. Rosenfeld added that these cases are the ones in which the marrow is primarily attacked as opposed to those in which the glands and spleen are chiefly affected. T. C. Janeway[3] brought forward a case only moderately progressive in type, and of three months' duration, yet of the white cells 98 per cent. were lymphocytes, and 87 per cent. of these were of the large variety, while the number of the red cells was normal. Thus it would seem that a hard line cannot be drawn between acute and chronic forms—indeed the writer had seen a case with numerous large cells which lasted 18 months.

**Pernicious Anæmia.**—H. C. Colman[4] divides the remedies into three classes—intestinal antiseptics, transfusion of blood, and substances stimulating the formation of blood. He finds the first gives only a temporary aid to tide over a crisis, and is sometimes harmful. At present no striking results have been obtained with antiseptics, though patients have shown improvement. On the other hand, out of 22 cases reported as cured 15 were treated with arsenic. It must often be pushed to the limit of safety, and continued for long periods, and should not be stopped suddenly, but gradually reduced if necessary. Vomiting and diarrhœa may be produced by the disease itself, and may even be relieved by arsenic, but bad results may undoubtedly be produced by it. Phosphorus is praised by Broadbent, and iron has been found of use when improvement has begun, but not during the periods of acute blood destruction, when it is apparently apt to do harm, and may irritate the alimentary canal and increase the diarrhœa. Dr. Colman has followed up the history of 22 cases which have been reported as cured of late years, and of these he finds that 9 died within four years, 7 had been lost sight of, 5 remained well for some years, and 1 for twelve years at least. The best results were obtained where patients continued to take small doses of arsenic for many years with occasional intervals. W. Hunter's treatment[5] comprises hygiene of the teeth and mouth ; lavage of the stomach, and the use of salicylic acid in three-grain doses ; intestinal antiseptics, *e.g.* $\beta$ naphthol, salol, perchloride of mercury, and rectal irrigation, a farinaceous diet, and lastly injections of anti-streptococcal serum. He[6] considers that the disease is preceded by long-continued septic trouble in the mouth, stomach or intestines, after which appears a sudden and profound anæmia, dependent on hæmolysis and accompanied by toxic symptoms, such as fever. There are definite lesions of the tongue and intestinal mucosa. Those on the tongue appear as a recurrent glossitis, producing sore, inflamed patches which leave the tongue smooth and glazed. Hunter[7] showed a patient in March apparently cured under his method. The man had suffered from alveolar abscess for ten years, and from recurrent attacks of gastric pain and glossitis, urobilinuria, tingling in the fingers and toes, pyrexia, great weakness and typical blood changes. The teeth were thoroughly treated, mercuric chloride was given regularly. Streptococcic serum in doses of 10, 10, and 5 c.c. was injected, and finally arsenic was given, with the result that the blood became normal and almost every trace of the disease vanished. He acknowledges that the real specific agent in the disease has not been discovered, though the streptococcus is associated with it in its action. The lesions in the alimentary tract may be compared with those in typhoid and the throat lesions in diphtheria. Here the special toxins are formed which produce the extreme blood destruction. Rumpf[8] finds that the blood during pernicious anæmia is extremely poor in potassium and rich in chlorides. He is inclined to think that the toxins have a special affinity for potash, and that it is the loss of this element which leads to the destruction of the red cells. He therefore gives large doses of various potash salts and finds that permanent cures in some cases are the result. W. Edgecombe[9] discusses a case of secondary anæmia becoming pernicious, which resembled some of Hunter's cases in its course. The patient was an anæmic lady who suffered for years from nasal suppuration and slight hæmorrhages, but both were very small in quantity though long continued. Finally the anæmia became profound, attacks of diarrhœa came on, retinal hæmorrhages, high-coloured urine, dyspnœa, and œdema were noticed. Great improvement followed the use of Hommel's hæmatogen and oxygen, but arsenic was not tolerated by the stomach. She then relapsed, and the hæmoglobin fell to 8 per cent., and the red cells to 485,000 before death. The case may be regarded either as a form of secondary anæmia, which, from repeated hæmorrhages, developed into the pernicious type, a process which is considered impossible by some writers; or else there was an infective condition set up by the constantly swallowed discharge from the nose. The close similarity of the blood state in some cases of simple and pernicious anæmia is well shown by R. Floyd and W. J. Gies.[10] In one patient, aged 19, the Hb. was only 12 per cent., the red cells 750,000, with much variation in size and shape, and there was an excess of lymphocytes. Under treatment with iron and a little arsenic, complete and permanent recovery took place. On the other hand, a patient of 64, very sallow in colour, who sank rapidly in spite of treatment showed Hb. equal 22 per cent., and the red cells 750,000, with other changes almost precisely similar to those in the first case. Vomiting and pyrexia here occurred in the last stage, and the disease seemed to be typical pernicious anæmia. These writers, like Hunter, insist on the need of taking clinical symptoms into account as well as blood analysis.

**Constitutio Lymphatica** is the name given to a state where there is hyperplasia of the lymph nodes, spleen, thymus, and marrow, with hypoplasia of the heart and aorta, and sometimes rickets. Sudden death is said to occur in these patients under various conditions. G. Peacocke[11] reports five cases where sudden death took place under slight illnesses, and changes in the lymphoid tissues were found in all. Most of the cases of death from an enlarged thymus have presented hypertrophy in other gland tissues such as Peyer's patches, and it is doubtful whether the enlarged thymus causes any mechanical hindrance to the circulation at all. The smallness of the heart is not universal, and the writer thinks it has no share in the fatal results. It is important to recognise the enlargement of the various glands, for many of the sudden deaths recorded in these patients have occurred during the administration of chloroform, or under the shock of a slight operation or of falls into the water. No detailed account of the blood appears to be on record.

1 Amer. Jour. Med. Sci., March. 2 Amer. Jour. Med. Sci., August. 3 Med. Rec., April 27. 4 Edin. Med. Jour., April. 5 Med. Chron., June. 6 West London Med. Jour., July. 7 Lancet, March 30. 8 Med. Rec., June 1. 9 Brit. Med. Jour., July 4. 10 Med. Rec., April 27. 11 Dublin Jour. Med. Sci., August

